# Predicting Gonadal Germ Cell Cancer in People with Disorders of Sex Development; Insights from Developmental Biology

**DOI:** 10.3390/ijms20205017

**Published:** 2019-10-10

**Authors:** Leendert H. J. Looijenga, Chia-Sui Kao, Muhammad T. Idrees

**Affiliations:** 1 Professor Translational Patho-Oncology, Department of Pathology, Lab. for Experimental Patho-Oncology, Erasmus MC-University Medical Center Rotterdam, and Group Looijenga, Princess Máxima Center for Pediatric Oncology, 3584 CS Utrecht, The Netherlands; 2 Department of Pathology, Stanford University School of Medicine, Stanford, CA 94305, USA; ckao2@stanford.edu; 3 Department of Pathology, Indiana University School of Medicine, Indianapolis, IN 46202, USA; midrees@iupui.edu

**Keywords:** germ cell cancer, developmental pathogenesis, individual risk assessment, prediction, disorders of sex development

## Abstract

The risk of gonadal germ cell cancer (GGCC) is increased in selective subgroups, amongst others, defined patients with disorders of sex development (DSD). The increased risk is due to the presence of part of the Y chromosome, i.e., GonadoBlastoma on Y chromosome GBY region, as well as anatomical localization and degree of testicularization and maturation of the gonad. The latter specifically relates to the germ cells present being at risk when blocked in an embryonic stage of development. GGCC originates from either germ cell neoplasia in situ (testicular environment) or gonadoblastoma (ovarian-like environment). These precursors are characterized by presence of the markers OCT3/4 (POU5F1), SOX17, NANOG, as well as TSPY, and cKIT and its ligand KITLG. One of the aims is to stratify individuals with an increased risk based on other parameters than histological investigation of a gonadal biopsy. These might include evaluation of defined susceptibility alleles, as identified by Genome Wide Association Studies, and detailed evaluation of the molecular mechanism underlying the DSD in the individual patient, combined with DNA, mRNA, and microRNA profiling of liquid biopsies. This review will discuss the current opportunities as well as limitations of available knowledge in the context of predicting the risk of GGCC in individual patients.

## 1. Introduction

Historically, germ cell tumors (GCTs) (independent of anatomical localization), were considered as a highly heterogeneous group of neoplasms, including both benign and malignant variants, comprising various histological elements in pure or mixed form. The pathological classifications differed among geographic areas and countries, and even within countries depending of the system followed [1]. This significantly hampered the exchange of relevant information, both related to clinical handling of the respective patients as well as performing informative (translational) research studies. Mixing various subtypes of testicular GCTs will underestimate the potentially relevant observations, both in a clinical as well as a laboratory setting. Therefore, an alternative classification system is deemed necessitous and would be highly beneficial. This process started in 2005 by the introduction of a developmental biology–based alternative classification model, taking into account their potential histological heterogeneity, predominantly fueled by knowledge of their (assumed) cell of origin [2,3]. The various GCT entities represent therefore defined, and well recognized from a developmental point of view, stages of germ cell maturation during physiological development, characterized by a set of (more or less) specific parameters. These include morphology, mRNA, microRNA, and protein profiles, as well as molecular genetic make-up (including epigenetics). Based on various independent confirmatory and multidisciplinary studies, this novel proposal was accepted unanimously at the last consensus meeting of the World Health Organization (WHO) held in 2015. It resulted in the current WHO classification (2016) [4,5], in which testicular GCTs (TGCTs) are subdivided into two main variants. These include the non-GCNIS-related and the GCNIS-related GCTs, also specifically summarized recently [3,6]. It must be kept in mind that the non-GCNIS-related GCTs encompass in fact two different types of TGCTs, being the pediatric teratomas/yolk sac tumors (Type I) and spermatocytic tumors (Type III) by virtue of having different cells of origin and pathogenesis. Therefore, they must not be mixed in the context of both clinical as well as laboratory studies.

The parameters related to cellular and histological composition, mRNA, microRNA and protein profiles, and genomic constitutions, are instrumental to recognize these various types, and as such crucial for diagnostic purposes. In addition, the parameters are instructive for both understanding their pathogenesis (i.e., cell of origin and initiating events), as well as progression related phenomena. Therefore, they will be highlighted hereunder, with the focus on those related to patients with disorders of sex development (DSD). Recognition of this overarching classification system resulted in identifying a well-defined set of biomarkers for primary (testicular) diagnosis as well as of metastatic and relapsed lesions.

## 2. Classification of GCNIS- and Non-GCNIS-Related Testicular GCTs

**Figure 1:**
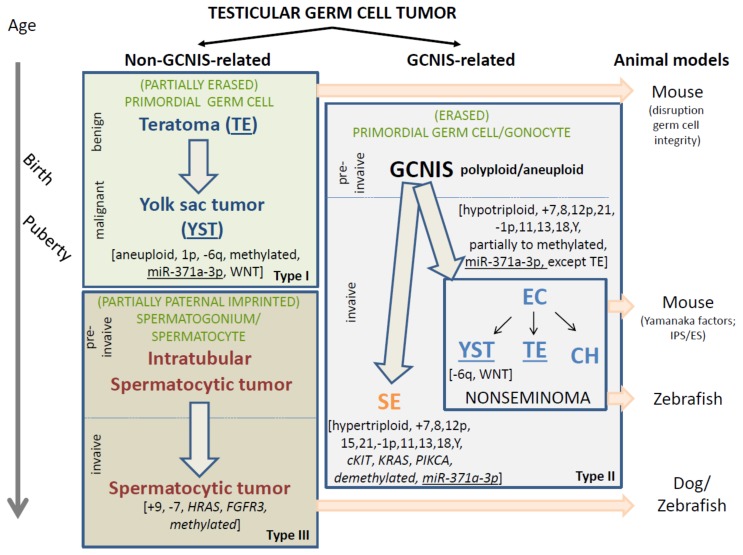
Schematic representation of the various entities of testicular germ cell tumors (GCTs). The time line is indicated on the left side and the proposed animal models on the right. The GCTs include the non-GCNIS (germ cell neoplasia in situ) related GCTs (left panel) and GCNIS-related GCTs (right panel). The non-GCNIS related GCTs are subcategorized into the prepubertal teratomas (TE) and yolk sac tumors (YST) as well as the spermatocytic tumors. These are also referred to as Type I and III, respectively. The GCNIS-related GCTs are histologically (and clinically) subdivided into the seminomas (SE) and the various elements of nonseminomatous GCTs, being embryonal carcinoma (EC), YST, choriocarcinoma, and TE. Note the overlapping histology between the prepubertal TE/YST and the TE and YST elements in the GCNIS-related nonseminomas. However, they have a separate (and independent) pathogenesis (see text for further details). The presumed cells of origin are indicated in green, reflecting a (partially and fully erased) primordial germ cell (Type I and II), to partially paternal imprinted spermatogonium/spermatocyte (Type III). The precursors are indicated when known (preinvasive), while specifically the benign and malignant behavior of the pediatric TE and YST is highlighted. In addition, the most prominent and recurrent molecular genetic changes are indicated, of putative interest to be used for molecular pathological approaches. These include total genomic anomalies, like polyploid/aneuploid, specific chromosomal imbalances like losses (-) and gains (+), as well as recurrent mutations (*italics*). In addition, the methylation status is indicated as well as the possible use of miR-371a-3p as a liquid biopsy molecular biomarker (*underlined*). All malignant histological elements, independent of age, are identified by this biomarker (except TE). The WNT pathway is specifically involved in the YST components, independent of age and also of pathogenesis.

The types of testicular GCTs are divided into two main categories (Figure 1). The Type I and III testicular GCTs are together referred to as non-GCNIS-related GCTs. The Type II testicular GCTs are referred to as GCNIS-related GCTs. This is simply based on the recognition of the different cells of origin and related pathogenesis, in which the knowledge on the origin of the Type II tumors (i.e., GCNIS, see below), is the dominant player in the classification because of its well-recognized status. This distinction between the GCNIS-related and non-GCNIS-related testicular GCTs is of relevance because of their different clinical behavior, i.e., malignant versus (predominantly) benign. As such, they will be discussed separately. Of special notion is the fact that, so far, morphology, mRNA, microRNA, and protein profiles of (Type I and II) teratoma and yolk sac tumor elements are similar; therefore, noninformative to make a differential diagnosis. However, this is consistently the case regarding their molecular genetic make-up, being therefore of diagnostic value. In addition, various animal models have been reported to be informative for GCT, which will be summarized hereunder because of their potential impact in understanding the pathogenesis of this type of cancer (see also Figure 1).

## 3. Spontaneous and Laboratory-Generated GCT Animal Models

Final elucidation of the pathogenetic mechanisms of the various types of testicular GCTs, especially the earliest events involved, might be dependent on animal models (both spontaneous as well as laboratory-induced). For the non-GCNIS-related testicular GCTs (i.e., prepubertal teratomas/yolk sac tumors, Type I), various animal models have been proposed, both spontaneously occurring as well as generated by genetic modification, especially in mice [7]. This includes the prone strain of mice (129J) that has been explained based on a dnd inactivating mutation [8]. Of interest is that a pleiotropy of genes disrupted in the embryonic germ cell lineage result in Type I-like teratomas (prepubertal type), including *p53*, *pten*, and *ras* [9,10], while formation of yolk sac tumor has been only reported rarely [11]. The likely spontaneous animal model for the other variant of non-GCNIS-related GCT (spermatocytic tumor; Type III testicular GCTs) is the dog [12], while also a genetically modified mouse model has been described, although being presented as mimicking seminoma (i.e., Type II) [13,14]. In addition, another laboratory generated mouse model has been reported for the pediatric (prepubertal type I) teratoma/yolk sac tumor. It is generated by combined forced expression of inactivated *p53*, *myc*, and oncogenic *hras*, resulting in tumors originating from embryonic like stem cells. In addition, this study also demonstrated that using the pluripotency factors, *oct3/4*, *klf4*, *myc*, and *sox2* (also known as the Yamanaka factors, found to be diagnostically of relevance (as discussed in the section: immunohistochemistry GCNIS-related (Type II) GCTs), a pluripotent GCT was generated [15]. Most likely, the model being most similar to the human Type II GCTs is the mouse model elegantly generated using a germ cell specific activated *kras* and inactivation of *pten* [16]. The model generates malignant tumors composed of both embryonal carcinoma as well as teratoma, including metastatic capacity of the first. Moreover, the model supports the window of sensitivity to initiate these GCTs during prenatal development. It is tempting to speculate that this mimics the GCNIS-related GCTs, although confirmation is needed. In addition, two zebrafish models have been reported, seemingly informative for the pathogenesis of GCNIS-related GCTs, mainly seminoma (and possibly spermatocytic tumor) (also discussed below). These include the *alk6b* impaired model [17,18] as well as *lrrc50* [19]. It remains so far to be proven what the actual relevance of these models is for the human pathologies observed, although they deserve further investigation.

## 4. Non-GCNIS-Related (Types I and III) Testicular GCTs: Cells of Origin

This category (to be discussed below in detail) includes pediatric (Type I) testicular GCTs, histologically composed of either teratomas and/or yolk sac tumor, predominantly diagnosed at early (pediatric) age. However, it is strongly recommended that the term “pediatric” GCTs is prevented as much as possible, because it will by definition result in a mixed population of these kind of tumors as well as GCNIS-related (Type II) GCTs, as recently demonstrated in a molecular study related to epigenetics (i.e., DNA methylation), clearly separating the two different entities of GCTs [20]. This mixture will dilute potentially relevant findings. The cell of origin of pediatric testicular teratoma/yolk sac tumor is still largely unidentified, although an early embryonic germ cell is most likely [21]. The other non-GCNIS related GCT is the so-called spermatocytic tumor (previously spermatocytic seminoma) (Type III testicular GCT). This is a well-defined entity only diagnosed in the post-pubertal testis, although sometimes misdiagnosed as seminoma. This variant originates from a more mature germ cell compared to the other GCTs, being either a spermatogonium or spermatocyte.

### 4.1. Prepubertal-Type Teratoma and/or Yolk Sac Tumors (Type I Testicular GCT)

As indicated, the prepubertal-type teratoma and/or yolk sac tumor (Type I GCTs) are predominantly diagnosed at an early age [22,23,24,25,26]. They can also occur later in life, although exceptionally [27,28]. Histologically these (Type I GCTs) can be composed of only two elements, either teratoma (representing potentially all germ layers, i.e., endo- ecto-, and mesoderm) and/or yolk sac tumor. The yolk sac tumor element progresses from the teratoma, representing the transition from benign to malignant. This is completely different from the pathogenesis of the GCNIS-related GCTs, in which the teratoma element(s) originate from an embryonal stem cell component, being embryonal carcinoma (to be discussed in detail later). Though morphologic features may be helpful, in a histologically pure teratoma, there are in principle two informative ways to investigate its malignant potential (i.e., behavior). This is based on demonstration of the presence (i.e., malignant) or absence (potential benign) of GCNIS (see below under Type II testicular GCTs) in the adjacent parenchyma. In the case of only atrophic seminiferous tubules, the absence of GCNIS must be interpreted with caution. In those cases, investigation of the genomic composition (i.e., diploid versus aneuploid) is much more informative. In clinical practice, complete surgical removal (which is often the case in the testis) and presence of yolk sac tumor elements are the major clinical parameters predicting clinical behavior [29]. The general rule can be applied that if other histological elements are present apart from teratoma and yolk sac tumor, i.e., seminoma, embryonal carcinoma, or choriocarcinoma (in relation to their specific immunohistochemical profiles), the tumor must by definition be classified as a GCNIS derived (Type II), and as such be considered as malignant (see Figure 1 for decision making).

#### 4.1.1. Risk Factors

Apart from familial predisposition, no other risk factors have been reported for this type of testicular GCTs.

#### 4.1.2. Immunohistochemistry

Various immunohistochemical staining patterns have been reported to be informative for the identification of the various elements of teratoma, although none have a proven impact on prediction of malignant behavior, i.e., potential of yolk sac tumor formation. In fact, they are identical to their counterparts in GCNIS-related testicular GCTs and will be discussed below. In contrast, detection of Alpha Fetoprotein (AFP) and Glypican 3 is informative to detect presence of a yolk sac component, although not being absolute. Indeed, both false positive and negative findings are reported [5,30]. Considering the limitation of the immunohistochemical markers, evaluation of the molecular genetic composition is in principle more informative.

#### 4.1.3. Molecular Genetic Constitution

The prepubertal teratomas, independent of the level of maturity as well as histological composition (ecto-, endo-, and mesoderm) are diploid (46,XY) without recurrent somatic mutations [31,32,33]. No mutations have been identified in the assumed candidate gene DND, based on mouse studies so far [34]. In contrast, the pediatric yolk sac tumors are always aneuploid, having defined chromosomal gains and losses. These relate to chromosome 1 (gain), 6q (loss), and part of 12p (gain, in particularly 12p13) [33,35]. The genes proposed to be involved are *STELLA*, *NANOG*, and *GDF3*. This is a relevant observation and must be kept in mind by interpreting (fluorescent) in situ hybridization ((F)ISH) data in the context of distinguishing a non-GCNIS- and GCNIS-related yolk sac tumor [36,37]. In fact, the method to be applied is a significant relevance, based on selected probes using FISH, or more broad copy number variations (CNV). In addition, loss of 6q is also found (specifically) in GCNIS-related yolk sac tumors, suggesting that it is related to formation of this specific differentiation lineage [38,39].

Apart from FISH, other molecular assays can be applied to detect CNV, including targeted PCR bases assays, single nucleotide polymorphism (SNP)- or DNA methylation-based arrays GCTs (450K and EPIC, for example) [20,39,40,41]. In addition to tumor specific molecular genetic changes, it has been identified that a number of SNPs are related to development of pediatric (including testicular) GCTs (of various anatomical localizations). These variants are present in the constitution DNA of the patient, and as such have to considered as susceptibility alleles, likely in interaction with environmental factors [42,43]. They are likely related, amongst others, to the targets BAK1 and SPRY4, interestingly involved in regulation of apoptosis of an embryonic germ cell. It remains to be determined what the exact impact of this observation is for the pediatric testicular teratomas/yolk sac tumors specifically. No genome-wide studies on the presence of somatic mutations have been reported so far. The selected studies all indicate that mutations are in fact rare, fitting with an embryonic germ cell lineage origin, allowing little-to-no mutations to be transferred to the next generation [44,45,46]. Progression of the teratoma elements to so-called somatic type malignancy might occur, showing the same genetic anomalies [47,48]. WNT signaling has been specifically identified in yolk sac tumor, including GCNIS-related subtype [49,50]. This might be related to the induction of cisplatin resistance [51,52].

### 4.2. Spermatocytic Tumors (Type III Testicular GCT)

The spermatocytic (Type III) GCTs were historically diagnosed as spermatocytic seminoma, based on the assumed similarities to seminoma [53], now simply renamed as spermatocytic tumor in the WHO 2016 classification system [5]. They are predominantly found in elderly men and their pathogenesis has been elucidated in large detail [53,54,55,56,57,58,59,60,61,62,63,64,65,66,67,68]. The cell of origin is either a spermatogonium or spermatocyte, in line with their cellular composition, RNA, and protein profile. They classically are composed of three cell types, being small, intermediate, and large. The precursor is known as intratubular spermatocytic tumor, filling up the seminiferous tubules, pushing the sertoli cells outwards, in contrast to the situation found in case of GCNIS. Clinically, spermatocytic tumors are indolent, although bilateral occurrence and very rare progression to sarcoma must be kept in mind [69,70]. The mechanistic explanation for these observations are lacking so far, although assuming spermatocytic tumors being only a hyperproliferative lesion, of which its molecular basis is possibly more systemic than (tumor) cell specific is tempting. In addition, intermediate characteristics between the Type II and III GCTs, as proposed in the most update classification [3], is a possibility as well. The answers to these questions will be obtained through detailed investigations of the cellular as well as molecular makeup of these unique cases in the context of the most recent classification.

#### 4.2.1. Risk Factors

So far, no risk factors have been reported for spermatocytic tumors.

#### 4.2.2. Immunohistochemistry

Various proteins have been identified to be informative for the diagnosis of spermatocytic tumors, especially in a comparative set up with seminoma (see below). These include XPA, CYP1, SSX2-4, as well as DMRT1 [71], CHK2, P53, p16INK4d and MAGE-4A [72], OCT2 and SAGE1 [73], NUT, and GAGE7 and NY-ESO-12 [66], being so-called testis-cancer-antigens. Of specific interest is DMRT1 [74]. This gene is mapped to chromosome 9, of specific relevance for spermatocytic tumors because of its consistent gain (discussed in the next paragraph). Most recently, it has been suggested that two variants of spermatocytic tumors exist, defined by specific protein profiles, and related to absence and presence of defined somatic mutations [63,65,68].

#### 4.2.3. Molecular Genetic Constitution

The spermatocytic tumors have a unique chromosomal constitution [53,57,61,63,65,68,75]. All investigated cases so far show additional copies (i.e., gain) of chromosome 9, being of diagnostic relevance. In fact, no changes have been reported in pediatric teratomas/yolk sac tumors for this chromosome, while loss is predominantly found in the GCNIS-related GCTs [39]. The candidate gene might be DMRT1, located in the short arm of chromosome 9, found to be amplified in a unique case, while expression is found in all [61]. Overall, spermatocytic tumors hardly show chromosomal breakage, resulting in sub-chromosomal gain and losses, although apart from gain of chromosome 9, loss of chromosome 7 was predominantly found [65,68]. The genes suggested to be involved are, apart from *DMRT1*, also *SOHLH1*, *DNMT3b*, *CTCFL/BORIS*, and *STRA7* (on chromosome 7). In addition, they hardy show somatic mutations, with the exception of two, being HRAS and FGFR3. Of specific notion is that these mutations can be found in sperm of elderly males, related to development of defined syndrome in the offspring referred to as selfish spermatogonial selection [65,76,77,78]. In conclusion, diagnostic distinction between spermatocytic tumor and seminoma (see below) can be accomplished using immunohistochemistry (preferentially using OCT3/4 and DMRT1) as well as molecularly (using FISH or CNV-based differences, focusing predominantly on chromosome 9 and 12p).

### 4.3. GCNIS-Related Testicular GCTs: Type II—Histological Diversity and Cell of Origin

**Figure 2:**
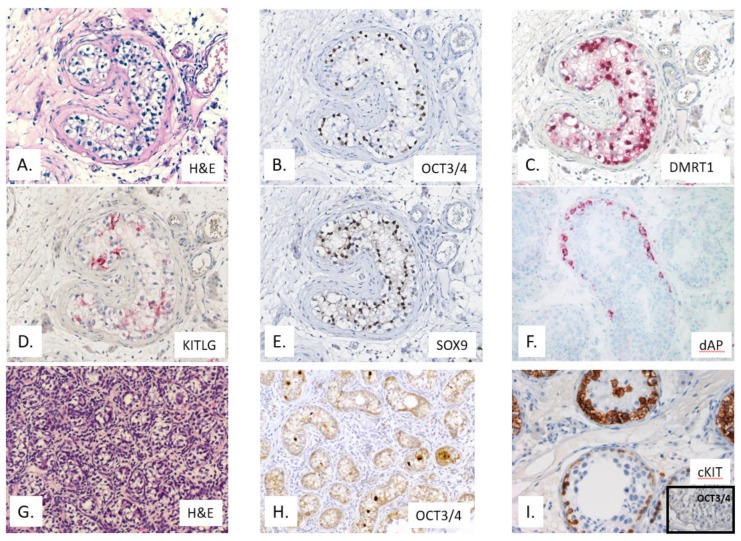
Representative examples of germ cell neoplasia in situ (GCNIS) (top two rows) (patient 30 years of age with a pure seminoma and GCNIS) stained using Hematoxylin & Eosin (H&E) (**A**) and immunohistochemistry for OCT3/4 (**B**), TSPY(**C**), KITLG (**D**), SOX9 (Sertoli cell marker) (**E**), as well as direct alkaline phosphatase (dAP) (**F**). In addition, the lower row shows a prepubertal testis with delayed maturation (H&E (**G**) and OCT3/4 positive (**H**)), as well as the “false” positive staining for KIT in normal spermatogonia (being OCT3/4 negative) (**I**). Multiplication 200×, except for **G**,**H** 100×.

All GCNIS-related testicular GCTs (also referred to as Type II GCTs) originate from a single precursor lesion, known as germ cell neoplasia in situ (GCNIS) according to the latest WHO classification [5], previously referred to as carcinoma in situ (CIS) [79], intratubular germ cell neoplasia, unclassified (IGCNU), or testicular intratubular neoplasia (TIN) [80] (Figure 2). The GCNIS cells represent an embryonic germ cell (i.e., primordial germ cells/gonocytes), characterized by several characteristics. The cells are in principle totipotent (omnipotent), and able to generate all differentiation lineages as can be found during embryonal development, both somatic (teratoma, including all three germ layers) as well as extra embryonic (yolk sac tumor and choriocarcinoma). Moreover, the germ cell lineage itself can be re-initiated in non-seminomas [81], representing the circle of life in full perspective, i.e., demonstrating its omnipotent character. Clinically, GCNIS-related testicular GCTs are categorized into seminomas and non-seminomas. While the former shows a rather homogeneous composition, representing in fact invasive GCNIS-like cells, the latter can contain all histological elements as found during physiological intra-uterine development, originating from the stem cell component embryonal carcinoma (representing embryonic stem cells). About 50% of GCNIS progress to seminoma and the other to non-seminomas.

**Figure 3:**
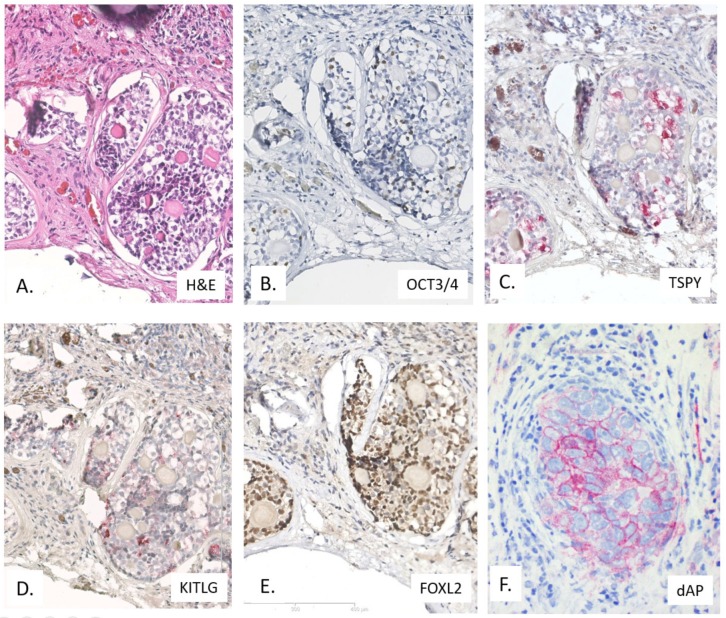
Representative examples of gonadoblastoma (GB) stained using Hematoxylin & Eosin (H&E) (**A**) and immunohistochemistry for OCT3/4 (**B**), TSPY (**C**), KITLG (**D**), FOXL2 (granulosa cell marker) (**E**), as well as direct alkaline phosphatase (dAP) (**F**). Multiplication 200×.

Although GCNIS is the proven testicular precursor of all Type II GCTs, the potential origin from gonadoblastoma (GB), albeit rare, must be considered, especially in the context of dysgenetic gonads [82,83,84,85,86,87]. This lesion is also composed of embryonic germ cells (like GCNIS, being OCT3/4 positive, see below), but now in the context of granulosa cells (being FOXL2 positive) instead of sertoli cells (SOX9 positive) (see Figure 3) [85,88]. It must be recognized that SOX9 can also be positive in rete testis, epididymis, and ductus deferens. GB must be clearly distinguished from intratubular seminoma based on the potential risk of a contralateral GCNIS-related GCT in these patients, related to one of the major risk factors for Type II GCTs, being disorders of sex development (DSD). Therefore, if a GB is diagnosed it must activate the clinical protocol for the patient assuming an underlying DSD [89]. This includes standardized pathological examination of the contralateral gonadal tissue (either biopsy or orchiectomy) [90]. Except in case of a retroperitoneal localization of a (assumed) GCT, the sole localization in the mediastinum is not indicative to exclude the presence of a testicular origin, i.e., demonstration of the presence of GCNIS, although they will contain gain of 12p if they are of the GCNIS-related like variant (Type II) [91,92].

#### 4.3.1. Risk Factors

The GCNIS-related (testicular) GCTs are known for the presence of a number of well-recognized risk factors, including cryptorchidism, in-/subfertility, familial predisposition, birth weight, and possibly hypospadias [6,87,93]. In addition, the aforementioned DSD has been found to be a major risk factor for this type of GCT [82,94,95,96,97,98,99,100,101]. Of additional relevance is the fact that these GCTs are predominantly found in young males between 20 and 45 years of age of a European descent, likely related to defined SNPs (discussed in detail below).

#### 4.3.2. Immunohistochemistry

GCNIS (as well as GB) cells show a consistent demethylated genome, detectable by immunohistochemistry [102,103,104], as well as a defined expression profile, both related to mRNA, microRNA, as well as proteins [21,105,106]. The profiles indeed mimic primordial germ cells/gonocytes, amongst others highlighted by the expression of OCT3/4, also known as OCT3 or OCT4 or POU5F1 [107,108,109]. The use of Bouin of Stieve’s fixatives might result in suboptimal and even false negative findings. In addition, these cells are also positive for PLAP (placental-like alkaline phosphatase), and cKIT, although the latter can result in overdiagnosis because of low expression in normal spermatogonia [110] (see Figure 2). In addition, they have a high glycogen content, to be detected using PAS. Moreover, the PLAP expression can be visualized using the direct enzymatic staining method [111]. Representative examples of the in the 2016 WHO-included diagnostic markers are given in Figure 2 and Figure 3.

Possible overdiagnosis at early age, in particular in the first year of postnatal life, for example in case of cryptorchidism repair surgery or DSD, is of specific relevance, for which the markers KITLG (stem cell factor) as well as TSPY (Testis Specific Protein on the Y chromosome) is of interest to be applied [90,112]. The various histological elements of invasive GCTs can be diagnosed using a number of informative proteins. The most relevant and informative are the (nuclear) transcription factors SOX17 and SOX2 to identify, in combination with OCT3/4, seminoma as well as embryonal carcinoma [113]. The yolk sac tumor and choriocarcinoma elements can be detected using AFP and hCG (human ChorioGonadotropin), respectively, although SALL4 and Glypican 4 are informative as well. Loss of PTEN has been reported to be related to the transition from GCNIS to an invasive GCT [114].

#### 4.3.3. Molecular Genetic Constitution

The finding on KITLG (see above) as one of the earliest changes in the transition from a primordial germ cell/gonocyte to pre-GCNIS [90] is, apart from diagnostic value, also relevant in the context of pathogenesis. It interestingly links the identification of specific single nucleotide polymorphisms (SNPs) associations to development of GCNIS-related testicular GCTs. Genome wide association studies (GWAS) demonstrate a reported link between the susceptibility SNPs and the KIT pathway related to primordial germ cell migration, survival, and proliferation [115,116,117,118,119,120]. Moreover, the other identified pathways are centrosome cycle, in line with other independently generated datasets [121]. The additional link to sex determination is obvious, based on DSD being one of the main risk factors, as well as GCNIS and GB being precursors. Moreover, the reported link to apoptosis (via CHEK2, GSPT1, and BRCA1) and DNA damage repair (RAD51 and BRCA1, amongst others) is of particular interest based on the mutational signature reported (see below). In addition, relevant variants, not identified by GWAS studies, have been identified based on a previously reported (*lrrc50*) zebrafish model, being related to ciliary function, found to be significant in GCNIS-related testicular GCTs, of which so far it is not clear whether it is related directly at the germ cell itself, or is related to the microenvironment [19,122]. In fact, to date it is not clear whether all variants act similarly during the pathogenesis of the GCNIS-related GCTs, or if some act early (for example during formation of GCNIS) and others late (for example progression to invasiveness). Such a heterogeneity might be expected based on targeted analyses of variants in patients with DSD [123].

The vast majority of histological elements of GCNIS-related testicular GCTs are characterized by extra copies (overrepresentation) of the short arm of chromosome 12, mostly as isochromosome 12p (i12p) [124,125,126,127,128]. This CNV is absent in GCNIS, and it is related to invasive growth [129]. This has been independently confirmed most recently based on purified GCNIS subpopulations [130]. Apart from gain of 12p, a number of tumors, predominantly seminomas, show high level amplification on specific subregions of 12, including *KRAS* [127,131,132,133,134]. While still a number of GCNIS-related GCTs are reported without gain of 12p, predominantly seminomas [39,135], and are suggested to have a (slightly) different pathogenesis (with preferential *cKIT* mutations), which is of relevance to keep in mind in the context of molecular pathology. In addition, it might be of specific relevance to investigate these cases to identify the important genes on 12p in more detail. It supported the model that polyploidization is one of the first steps in the formation of GCNIS. The study in addition provided strong indications that most of the GCNIS cells (without gain of 12p or *cKIT* mutations) will not progress to an invasive GCT. In that context it is relevant to state that so far it is not proven that all GCNIS will progress to full blown cancer, of relevance for screening purposes. Gain of 12p can be detected using various approaches, including FISH, SNP array, as well as the methylation array (450K or EPIC) [37,39,40,124,127,132,134,136]. For these later approaches, the presence of sufficient amount of tumor cells is a logical prerequisite for proper interpretation.

Regarding mutational status, a number of targeted and high throughput studies have been reported [117,137,138,139]. The most extensive multidisciplinary study on GCNIS-related testicular GCT was recently reported by Shen et al. [135]. Overall, the results are in the same direction. They, independent of histological composition, have a very low frequency of somatic mutations of about ~ 0.1–0.5 per Mb, with only a few exceptions, being in the same range as pediatric (non-GCNIS) cancer as well as spermatocytic tumors. It is likely that this is related to the evolutionary mechanism preventing transmission of (harmful) genetic changes to the next generation. Based on CNV and mutational status, two variants of seminomas do seem to exist, one with and one without KIT mutations, whereby the wild type variants do show a higher level of genome methylation. In addition, there is likely a less prominent presence of gain of 12p in the *cKIT* mutated seminomas. The data supporting this hypothesis are obtained both from intracranial cases [140] as well as of the post pubertal testis [135]. The results indicate that, although recurrent, these two mechanisms are seemingly partly overlapping. A dedicated study on this specific finding would be of significance, because it might reveal the possible responsible genes on 12p related to progression from GCNIS to invasive growth [129]. In this context, it is of relevance to indicate that the largest DNA methylation series of testicular Type II GCTs excludes those lacking gain of 12p [39]. Specific reanalysis of these cases might therefore be of specific interest.

Apart from *cKIT*, mutations, or amplification [141,142], only *KRAS*, *NRAS*, and to (even) a lower extent *PI3CA*, seem to be affected by mutations. In the context of the cKIT-KITLG loop, either autocrine or paracrine, the loss of KITLG in non-seminomas is of interest [143]. CNV seems to be more profound, including of course *KRAS* (being on 12p). In addition, amplification of *MDM2* was also found, being in accordance to earlier findings, showing that the P53/MDM2 axis is involved in therapy resistance [144,145]. In addition, other possible targets are identified as well, being *RAC1* and *FAT1*, possibly used for targeted therapy in some cases. [146,147]. In this context, a more targeted therapy can be applied [146]. In addition, microsatellite instability has been reported to be involved with cisplatin resistance [148], although not identified in the largest series investigated, possibly due to bias to mainly treatment sensitive cases [135]. The only study that included the matched GCNIS to the invasive non-seminoma mutations status demonstrated that none of the mutations are identified in the precursor lesion GCNIS, while being already aneuploid [130]. This indicates that the mutational load, although overall low, is related to progression and is also found to be heterogenous within the primary tumors, possibly even absent, while most likely related to therapy resistance at metastatic sites.

The results obtained so far support the model that GCNIS-related testicular GCTs are in fact developmental cancers, in which a disturbed microenvironment, possibly initiated by the supportive cells, resulting in an inappropriate niche for the gonadal embryonic germ cells for proper maturation is one the major risk factors for the development of this cancer [2,6,21,59,149,150,151,152,153,154]. This must be kept in mind related to optimal diagnosis. In other words, no highly informative molecular biomarkers are identified except CNV (i.p. gain of 12p), as well as mutations in a limited number of genes (*K*- and *NRAS*, as well as *PI3CA*). However, more developmentally related parameters could be a target of interest, even as liquid biopsy molecular biomarkers. These include overall methylation status, for example demethylation of *DPP3A* [39], hypermethyation of *RASSF1A* [155], or alternatively X inactivation and related hypomethylation of the related *XIST* promotor, uniquely found in GCNIS related (Type II) testicular GCTs in males [156,157]. However, one of the major candidates, relatively close to be implemented in clinical practice for malignant GCTs, both pediatric yolk sac tumors and GCNIS-related GCTs (except teratoma) is detection of embryonic microRNAs, in particular miR-371a-3p.

## 5. Application of miR-371a-3p as Molecular Biomarker for Malignant GCTs in Liquid Biopsies

The field of analyses of the potential use of miR-371a-3p as molecular biomarker for GCTs started by the publication in 2006 [158]. That study demonstrated that the members of the miR-371-3 can function as an alternative mechanism for inactivation of the P53 pathway without inactivation mutations due to LATS2 interaction. This resulted in absence of cellular senescence, now reported to be the case for all three members [159]. Another putative target of interest in RAD51 is related to DNA damage response (see above). Expression of miR-371a-3p was found, both using a high throughput as well as a targeted approach, to be highly informative in identifying the malignant component of GCTs [160,161,162]. Subsequently, it was demonstrated that it can be detected in serum, plasma, as well as cerebrospinal fluids of patients with a malignant GCT (either GCNIS-related or non-related, Type I or II) as well as in appropriate mouse xenograft models [163,164,165,166,167,168,169,170,171,172,173,174,175]. The only known origin of physiological expression is in spermatogonia [176]. The results so far demonstrate that miR-371a-3p is significantly more informative compared to the golden standard AFP and hCG, because it is expressed in all malignant elements, except teratoma. An alternative miR, being miR-375, has been suggested to be informative for teratoma as well, although not proven so far [135].

## 6. Conclusions

A summary regarding the current knowledge on the classification and (potential) biomarkers of GCTs is represented in Figure 1. These specifically relate to the various types of testicular GCTs, being both variants in the non-GCNIS-related and GCNIS-related tumors. Especially, CNV are informative to distinguish the types besides histological composition. Specifically, gain of the short arm of chromosome 12 for the invasive GCNIS-related components, and gain of chromosome 9 for the spermatocytic tumors. In addition, aneuploid and loss of 6q in the prepubertal yolk sac tumor, in contrast to the prepubertal teratoma, while all GCNIS-related teratoma are aneuploid and mostly contain gain of 12p. The methylation as well as mutational profiles are less informative, based on the possible overlap as well as (overall) low frequencies, respectively. The GCNIS-related, i.e., Type II GCTs, by definition malignant, are relevant in the context of patients with DSD. Identification of the precursor lesion, based on a defined set of histology-based biomarkers, including OCT3/4, TSPY, and KITLG, is crucial for proper risk stratification. Of particular interest is the miR-371a-3p, found to be highly informative for all malignant GCT components, both in pediatric and (young) adult patients, suitable to be used as liquid-biopsy based molecular biomarker. It is expected that it will change clinical handling of patients with GCTs of the testis as well as other anatomical localizations (extra-cranial and cranial) drastically within the coming years. Based on the data available so far, it will outperform the currently used golden standards AFP and hCG. However, liquid biopsy-based informative biomarkers for the precursor lesions, i.e., GCNIS as well as GB, relevant for screening of DSD patients at risk are still lacking.

## Abbreviations

AFP	Alpha Fetoprotein
CIS	Carcinoma In Situ
CNV	copy number variations
DSD	Disorders of Sex Development
EC	embryonal carcinoma
(F)ISH	(Fluorescent) In Situ Hybridization
GB	Gonadoblastoma
GBY	Gonadoblastoma on the Y chromosome
GCNIS	Germ cell neoplasia in situ
GCTs	Germ Cell Tumors
GGCC	gonadal germ cell cancer
GWAS	Genome wide association studies
hCG	human ChorioGonadotropin
IGCNU	Intratubular Germ Cell Neoplasia, Unclassified
PLAP	Placental like alkaline phosphatase
SE	seminoma
SNP	Single Nucleotide Polymorphism
TE	teratoma
TSPY	Testis specific protein on the Y chromosome
TIN	Testicular Intratubular Neoplasia
YST	yolk sac tumor
WHO	World Health Organization
